# The DREAM BIG project as a model for harmonizing early measures of parental care and parent-child interactions across epidemiological cohorts

**DOI:** 10.3389/frcha.2023.1206922

**Published:** 2023-10-20

**Authors:** Eszter Szekely, David P. Laplante, Henning Tiemeier, Jonathan Evans, Rebecca M. Pearson, Mona Bekkhus, Marian Bakermans-Kranenburg, Marinus H. van IJzendoorn, Ashley Wazana

**Affiliations:** ^1^ Lady Davis Institute, The Jewish General Hospital, Montreal, QC, Canada; ^2^ Department of Psychiatry, McGill University, Montreal, QC, Canada; ^3^ Department of Social and Behavioral Science, Harvard T. H. Chan School of Public Health, Boston, MA, United States; ^4^ Department of Child and Adolescent Psychiatry and Psychology, Erasmus MC University Medical Center, Rotterdam, Netherlands; ^5^ Centre for Academic Mental Health, Population Health Sciences, Bristol Medical School, University of Bristol, Bristol, United Kingdom; ^6^ Department of Psychology, Manchester Metropolitan University, Manchester, United Kingdom; ^7^ Department of Psychology, University of Oslo, Oslo, Norway; ^8^ University Institute of Psychological, Social and Life Sciences, Lisbon, Portugal; ^9^ Psychiatry Monash Health, Monash University, Melbourne, VIC, Australia; ^10^ Research Department of Clinical, Educational and Health Psychology, UCL, London, United Kingdom

**Keywords:** maternal sensitivity, mother-child interactions, replication, developmental model, prenatal, genetic risk, data harmonization

## Abstract

Parenting is a key contributor to child development. The effects of parenting, however, also depend on child characteristics, including genetic factors. A more complete appraisal of the role of parenting thus requires a comprehensive developmental model which explores questions about parenting behavior, child susceptibility to parenting, and child psychopathology. Moving forward, we need to not only be concerned about sample sizes that limit testing of comprehensive models but also the need to replicate findings across multiple settings and samples. A consortium which harmonises key measures offers the opportunity to examine these questions. The Developmental Research in Environmental Adversity, Mental health, BIological susceptibility and Gender (DREAM BIG) consortium includes six international longitudinal prospective birth cohorts to explore the early life origins of major psychiatric disorders in childhood. Here, we will provide a brief overview of parental care research, methodological limitations, and two exciting recent attempts (i.e., the DREAM BIG consortium and the CATS-project), that address key methodological challenges.

## The context: the importance of parental care

Humans are among the most helpless of species at birth and they remain dependent on their parents for a long time before being able to navigate the world independently. Parental care has thus direct consequences for children's survival, growth, and psychosocial development (1). The early caregiving environment supplies young children with the necessary experiences and support for achieving their developmental milestones (2), and it plays a key role in shaping children's social-emotional and cognitive development (3). Established models of parenting postulate that the quality of the parent–child relationship is the integrated product of three broad factors: parental characteristics, infant characteristics, and context which may influence parenting in a supportive or stressful way (4, 5). Parent-infant interactions encompass a diverse range of dyadic processes, among which the most heavily investigated construct is *maternal sensitivity*, or the mother's ability to accurately perceive and interpret their infants' signals and respond to them in a prompt and appropriate manner (6–8). Decades of research on maternal sensitivity have provided evidence on its link with numerous domains of child development, including social adjustment (9), executive functioning (10), cognitive and language outcomes (11, 12), and, not the least, children's attachment relationships (13, 14).

### Environmental sensitivity

Children respond differentially to parenting though with genetic and prenatal environmental factors contributing to an increased sensitivity to the environment called postnatal plasticity (15). While Belsky documented that this plasticity (measured as temperament) emerges from genetic factors (differential susceptibility) (16, 17), and Boyce and Ellis posited that this susceptibility would be rather environmentally induced, (16), both show that plasticity factors influence how individuals interact with the environment. Three patterns of environmental sensitivity have been described in the literature: diathesis-stress, differential susceptibility, and vantage sensitivity (17, 18). In diathesis-stress, a biological marker represents a disadvantage in unfavourable environments in that outcomes for carriers of that marker can only be approaching the outcome levels of noncarriers if the individual is exposed to average or advantageous environments. In vantage sensitivity, the opposite is true: the biological marker represents an advantage over noncarriers, such that in unfavorable environments carriers and noncarriers develop similarly, while carriers show increasingly better outcomes as the environment becomes more advantageous. Finally, in differential susceptibility, highly susceptible children are more responsive to both adverse and supportive environments than non-susceptible children for better or worse (15).

### Gene-environment interplay

Surprisingly, few studies have examined the associations between genetic risk, prenatal adversity, and maternal care in predicting child psychological functioning, even though the quality of early parental care can be a crucial mitigating factor of the effect of prenatal environmental or genetic risk (19). In a three-way interaction model, we found that maternal looking away behaviour (negatively correlated with maternal sensitivity) moderates the risk associated with prenatal depression and the 5-HTTLPR genotype to predict depressive symptoms at 18 months, but not at 24 months (20). Similarly, we have reported that maternal looking away behaviour moderates the developmental risk from low birthweight and *DRD4* to predict disorganized attachment (21). These findings suggest that, in children with genetic and prenatal risk, the risk for psychopathology was attenuated when the mother looked away less frequently. These findings are in line with previous studies reporting that the frequency of self-reported maternal stroking during early infancy moderated the effect of pregnancy anxiety on internalizing problems when the children were 3.5 years of age (22). For mothers who experienced high levels of pregnancy-specific anxiety, high levels of postnatal stroking were related to lower internalizing scores in their children. Similar results of a moderating role of maternal stroking on child internalizing problems have also been reported for prenatal maternal depression (23) and general anxiety (24). Finally, in our recent study in the Maternal Adversity, Vulnerability and Neurodevelopment (MAVAN) sample, we found evidence for the presence of two-way interaction effects on toddler attention function, namely that positive maternal behaviors observed during mother-child interactions at 6 months postpartum mitigated the effects of both prenatal adversity and dopaminergic polygenic risk on toddler attention function (25). However, our sample was limited to find a significant three-way interaction between prenatal adversity, dopaminergic risk, and parenting behavior, and the above two-way interaction effects need to be replicated in independent samples.

### Epigenetic processes

Fresh perspectives in understanding the complexities of the parent-child dynamics are also offered by behavioral epigenetic studies, which posit that the quality of maternal care sets epigenetic processes (e.g., DNA methylation) in motion that may ultimately affect offspring psychological development through modifying expression of genes involved in behavioral and stress regulation (e.g., *NR3C1, BDNF, OXTR*) (26). For example, harsh parenting contributes to similar epigenetic modifications in the child as early adversity, potentially affecting cognitive and socioemotional development in childhood (27, 28) and attachment style in adulthood (29). Importantly, epigenetic modifications are also affected by positive parent-child interactions (30, 31), translating into “positive” epigenetic mechanisms, which may act as a protective mechanism in the face of adversity-related increased DNA methylation of genes involved in behavioral and stress regulation (32, 33).

## The problem: limitations of current parenting research

### The replication problem

The replication crisis in science (34) is exemplified in psychological research, where the replication rate of key experiments is just above 10% (35). One reason for the lack of replication is that cohorts do not always assess the same developmental constructs, and, even when they do, they often use different measures to assess them. In addition, without the initial registration of research hypotheses and analytic plans (e.g., in the Open Science Framework) of observational studies, akin to that found with randomized controlled trials (e.g., www.clinicaltrials.gov), it is not often clear which findings (*a priori* vs. *post hoc*) are most likely to be replicated. For clinical trials, there are broadly endorsed initiatives of prospective harmonization of outcome measures, such as the COMET initiative (https://www.comet-initiative.org/) and the CROWN initiative (http://www.crown-initiative.org/). For already existing observational studies, the retrospective harmonization of relevant predictor and outcome variables across cohorts with similar measures may prove essential in producing replicable and generalizable research findings. As well, pre-registering planned correlational analyses in intentional initiatives to replicate findings will make for more convincing results.

### Measurement error

Gathering detailed observational data on parent-child interaction in large epidemiological cohorts is costly and unfeasible. However, complex developmental models that account for the interplay of genetic and pre- and postnatal environmental influences are incomplete without including precise measures on the quality of parental care. Measurement error can reduce statistical power for detecting true interaction effects in complex developmental models, as it inflates the variance of the estimate of the interaction term, similarly to multi-collinearity and non-normal distribution of the interaction terms (36–38). Observational measures of mother-child interactions are thus strongly preferred to self-report measures of parenting in studying complex developmental models in longitudinal cohorts. Observational measures, due to their complexity and cost, restrict potential sizes of epidemiological developmental cohorts. Thus, the need for larger sample sizes and valid cross-study comparisons, has led to increased interest in co-analyzing already existing data across studies. However, heterogeneity in study design and measures collected limit our capacity to easily compare or integrate data across studies (maelstrom-research.org) (39).

### Small sample sizes

To be truly informative, birth cohorts, particularly those with genetic data, require large samples to test complex computational models of developmental trajectories (40, 41). While harmonization of key predictor and outcome variables across multiple birth cohort studies will greatly assist in overcoming the replication problem, *a priori* or *post-hoc* harmonization of parenting measures will also help overcome the problem of small sample sizes, that are typical of focus cohorts of large epidemiological samples, where observational measures of parenting are available (42). Combining focus cohorts from multiple large birth cohorts with harmonized parenting data can increase sample size to levels sufficient to conduct tests of complex models.

### Aim of the present paper

The present paper discusses the relevance of a key methodological concept (i.e., retrospective data harmonization), which can help mitigate some of the challenges inherent in replicating study findings involving observational parent-child interaction measures. More specifically, this paper offers two valuable approaches to researchers who are interested in retrospective harmonization and integration of parent-child interaction data across independent samples.

The first approach comes from our ongoing initiative, the DREAM BIG consortium, which performs cross-cohort retrospective data harmonization of key constructs relevant to probe complex models of child development (e.g., the prenatal environment, genetic susceptibility, child psychopathology, and early parental care). The second strategy, as used in the CATS-project, focuses on the initial stages of retrospective data harmonization of observed maternal sensitivity. This method first evaluates the theoretical constructs underlying the measures and then the measures themselves prior to the recoding of original values. The DREAM BIG and CATS approaches offer helpful analytical solutions for restructuring parent-child interaction data collected with different instruments across multiple studies to indicate a comparable construct.

## A proposed innovative solution: the DREAM BIG consortium as a model of cross-cohort data harmonization of child developmental constructs

The Developmental Research in Environmental Adversity, Mental health, BIological vulnerability, and Gender (DREAM BIG) research consortium was established in 2016 to examine, in a multi-site design, the developmental origins of major mental disorders (www.dreambigresearch.com). DREAM BIG includes six prospective prenatal cohorts: Avon Longitudinal Study of Parents and Children (ALSPAC, UK) (43); Generation R Study (GEN-R, Netherlands) (44); Maternal Adversity, Vulnerability and Neurodevelopment (MAVAN) project (Canada) (45); Mother, Father and Child Cohort (MoBa, Norway) (46); Prediction and prevention of preeclampsia and intrauterine growth restriction (PREDO) study (Finland) (47); and Growing Up in Singapore Towards Healthy Outcomes (GUSTO) cohort (Singapore) (48). These cohorts have comparable measures on prenatal adversity—including prenatal maternal psychopathology and prenatal environmental adversity—genetic data, observed and self-reported early parental care and parent-child interactions, and child psychopathology. Our work thus far supports the hypothesis that prenatal maternal psychopathology, social-environmental adversity, and child genetic susceptibility for multiple psychiatric disorders and psychological traits predict emerging general and internalizing (e.g., depression and anxiety) psychopathology in 4-to-8-year-olds (49, 50).

## Harmonization of major constructs within DREAM BIG

**Figure 1:**
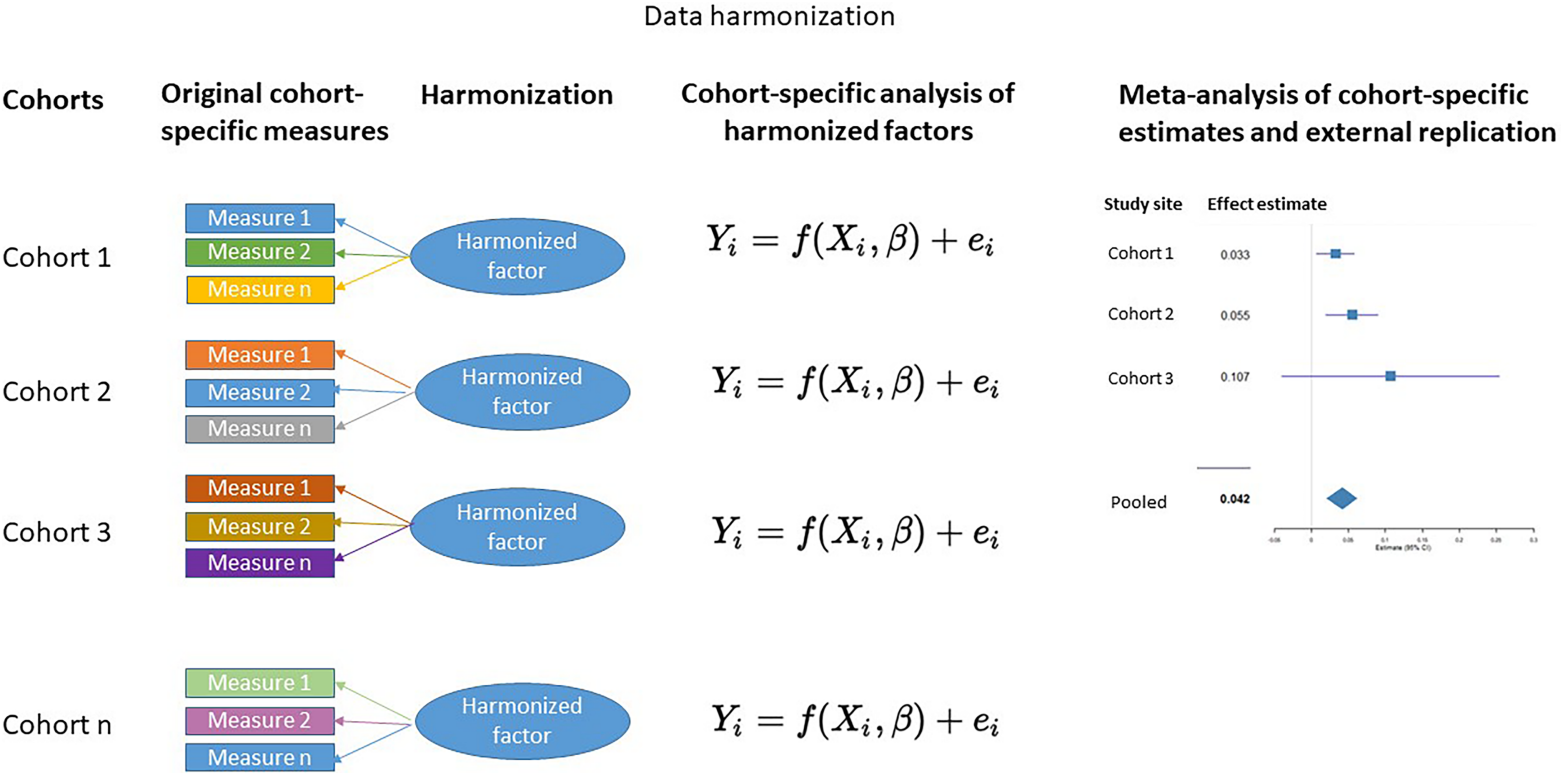
Final stages of retrospective data harmonization using the DREAM BIG approach.

To date, DREAM BIG has harmonized measures of prenatal adversity and genetic susceptibility, and created cross-diagnostic and hierarchical harmonized measures of child psychopathology by integrating information across multiple informants at multiple time points (Figure 1). A brief description of these measures are provided below and a summary of the main findings to date are presented in the Supplementary Material Table S1.

### Prenatal social-environmental adversity

This is a harmonized prenatal cumulative risk index derived from four major areas: stressful life events (i.e., death in family, accident, illness), contextual risks (i.e., poor housing conditions, financial problems), parental risks (i.e., alcohol and substance abuse, criminal involvement), and interpersonal risks (i.e., family conflict, domestic violence) (51, 52) using confirmatory factor analysis with a second-order hierarchical model.

### Prenatal maternal affective symptoms

This is a set of harmonized prenatal maternal psychological symptoms constructed using confirmatory factor analyses that identified a general prenatal affective symptoms factor and three specific factors: anxiety/depression; somatic symptoms; and pregnancy-specific anxieties across cohorts (50). These prenatal maternal affective symptoms factors predicted offspring psychopathology at age 4–8 years in a meta-analysis of three cohorts (50).

Both measures of adversity (i.e., prenatal social- environmental and maternal affective) build on previous successful harmonization initiatives between ALSPAC and Generation R (51, 52). DREAM BIG innovated by separating these two measures.

### Childhood psychopathology

This includes a harmonized, age-adjusted, general psychopathology factor (P-factor), and specific uncorrelated internalizing and externalizing factors consistent with the Hierarchical Taxonomy of Psychopathology (HiTOP) model (53), constructed using psychopathology measures rated by different informants (parent, child, teacher) at multiple time points between the ages of 4 and 8 years (54). A HiTOP approach to harmonizing psychopathology addresses important methodological concerns about diagnostic co-morbidity, homotypic and heterotypic discontinuities, and rater differences (53, 55).

### Genetic susceptibility

Polygenic scores (PGS) for internalizing (anxiety, depression), neurodevelopmental (ADHD, ASD), psychotic (schizophrenia, bipolar), and compulsive problems (anorexia nervosa, obsessive-compulsive, Tourette syndrome) will be computed in each cohort based on results of a Genomic Structural Equation Model (GenomicSEM) of 11 common psychiatric disorders using publicly available GWAS summary statistics (56). This approach models the structure of psychopathology at the genomic level and exploits genetic correlations between multiple psychiatric disorders modeled simultaneously. Further details about the GenomicSEM approach are available elsewhere (56).

### Maternal care

Four of the six DREAM BIG cohorts have observational measures available on maternal sensitivity and parent-child interactions. In MAVAN, maternal care and mother-child interactions were observed during free play using the Parent-Child Early Relational Assessment (PCERA) (57) at 6, 18, 36 and 60 months, the Ainsworth Maternal Sensitivity Scales (6) at 6 and 18 months, and the Behavioral Evaluation Strategies and Taxonomies (BEST) (Educational Consulting, Inc. Florida, US; S & K NorPark Computer Design, Toronto) at 6 months. In Generation R, maternal sensitivity was observed during free play using two subscales of the Ainsworth Maternal Sensitivity Scales at 14 months (sensitivity and cooperation), and during two structured mother-child interaction tasks using the revised Erickson 7-point rating scales for supportive presence and intrusiveness (58) at 36 and 48 months in a subsample (*n* = 1,079). In GUSTO, maternal sensitivity was observed during free play using the Revised Mini-A short form of the Maternal Behavioral Q-Sort-V (Mini-MBQS-V) (59) at 6 months, and during a structured mother-child interaction using the Erickson 7-point rating scale at 54 months. Finally, in a subsample (*N* = 1,240) of the ALSPAC cohort, the Mellow Parenting Observational System (60) was used to code mother-child interactions during the Thorpe Interaction Measure (61) at 12 months.

Our current work in DREAM BIG will also harmonize the measures of observed maternal sensitivity across cohorts to test for the presence of replicable two-way and three-way interactions between prenatal adversity, child genetic susceptibility, and early maternal sensitivity on the development of child mental health problems. Integrating both harmonized parenting and child measures allows for the inclusion of complex questions assessing a wide range of well-defined observable parental care measures.

## The CATS-project as a solution for the initial stages of retrospective harmonization of observed maternal sensitivity

**Figure 2:**
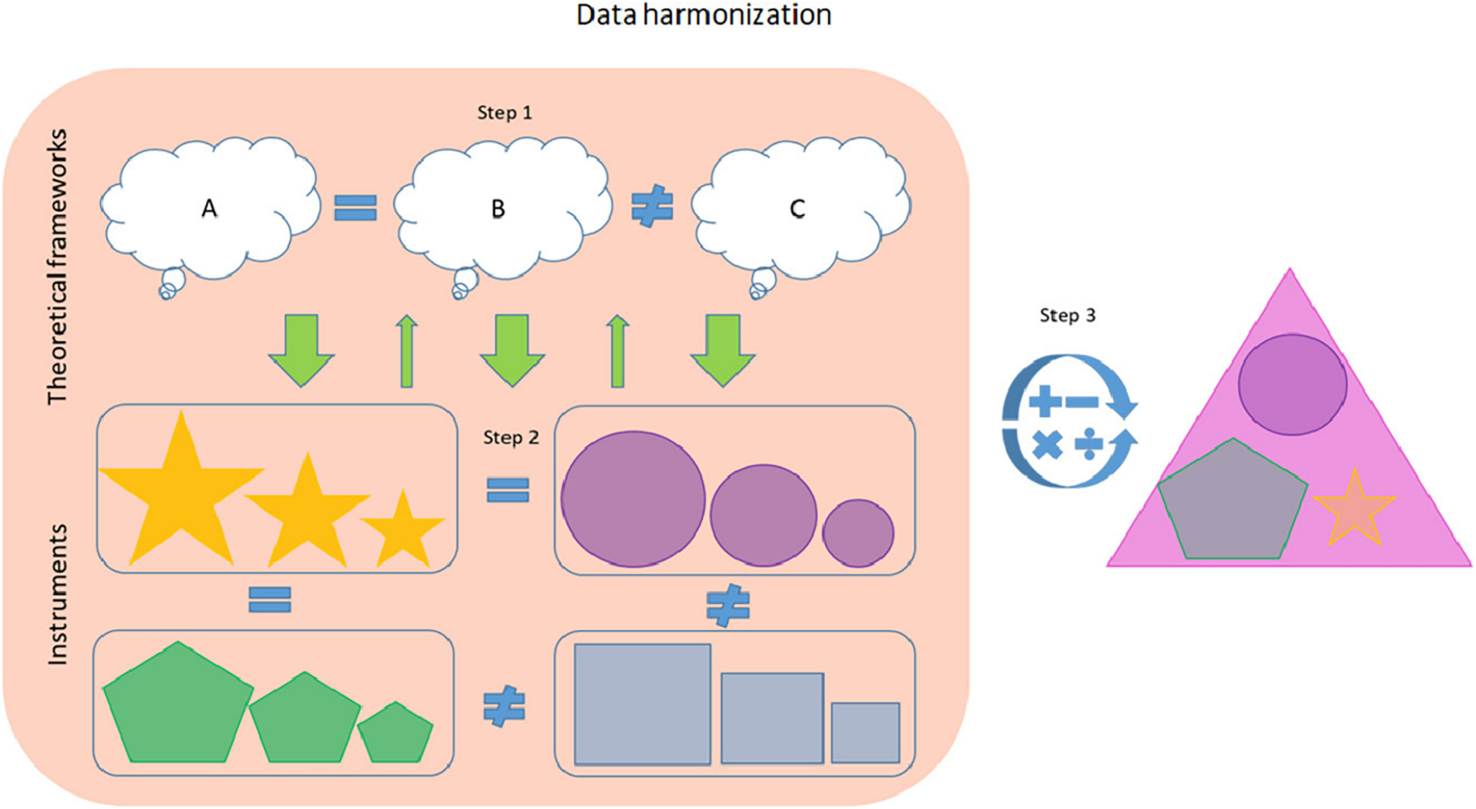
Initial stages of retrospective data harmonization using the CATS-project. Source: Graphical abstract from Verhage et al. (65).

Assessing the nature of dyadic dynamic processes, such as maternal sensitivity, is challenging, and brings critical attention to the core issue of assessment (26, 62, 63). The Collaboration on Attachment Synthesis CATS-project is a multi-site meta-analytic study focusing on synthesizing the literature regarding the association between parental cognitive representation of attachment and the child-parent attachment relationship (64). The strategy pertains to the early stages of data harmonization and can be applied to observational measures of maternal sensitivity. This three-step method includes a top-down approach to evaluating the theoretical constructs underlying the measures and a bottom-up approach to evaluating the measures prior to recoding the values (Figure 2) (64).

The first step represents a top-down strategy of defining a unitary construct by reviewing the existing literature screening for one or more dominant developmental framework(s). The second step is a bottom-up strategy whereby each instrument is evaluated against the theoretical frameworks identified in the first step. Here the researchers assess which theoretical subdimensions are measured by which subscale or item(s) and decide which (sub)scales or items to retain. The final step entails the recoding of scores to an identical metric based on the existing literature. So, in the case of parental sensitivity, the authors' search of the literature (step 1) indicated that the construct of parental sensitivity is derived from the attachment theory framework. The individual studies in the CATS database included eight different measures of parental sensitivity in total, which then needed to be evaluated (step 2) against the construct of maternal sensitivity derived in step 1. Finally, the authors recalculated the scores (step 3) of all the instruments to match the reference scale of one of the available instruments, the Ainsworth sensitivity scale, which is considered “gold standard” for measuring parental sensitivity (64). The authors recommend their method to be used in conjunction with the existing literature on the restructuring of measurements from different instruments into the same format (i.e., the later stages of the harmonization process) before analysis.

This exciting new strategy is filling a gap in the literature on data harmonization by providing researchers with a tool for pooling, amongst others, observational data on parental behaviors. However, this approach, which could be expanded to other predictor and/or outcome measures, is facilitated greatly by the development of new large scale epidemiological cohort studies in which predictor and outcome measures are harmonized before study onsets.

## Limitations

As inherent in all retrospective data harmonization techniques, the two approaches presented here are also subject to limitations including the complexity and necessity of expert domain knowledge to pool data, and the possibility that, despite best efforts, some data may not be comparable across cohorts due to, for instance, gross heterogeneity in measures. When the available data are not comparable, there is risk of data loss (66). Moreover, and also inherent to data harmonization, details with regard to the observational context can get lost. For example, parental sensitivity can be observed during free play, unstructured home observations, or stress-inducing tasks in a laboratory. Such observational contextual information can be included in analyses as potential moderating factors, but type of context and measure may be confounded when specific measures are only used in specific contexts.

## Implications

Harmonization and replication of complex models improve our ability to detect and understand methodologically robust and key nodes of environmental influences in children's social-emotional development. Given the rich primary and secondary intervention literature aimed at the modification of early parental care and parent-child interactions, more precise identification of susceptibility to and effects of these interactions, will additionally inform public health and primary care practices. For example, harmonized indicators of observed parent-child interaction may be used in the assessment, selection of target behaviors, intervention, and monitoring the effect of the intervention in the treatment of families with mental health problems (67).

## Conclusions

The CATS approach contributes to an important avenue of the initial stages of retrospective harmonization, while the DREAM BIG approach focuses on the later stages of retrospective data harmonization of developmental and parenting research to overcome the replication problem. Both approaches can be applied, in a complementary manner, to the cross-cohort harmonization of observed mother-child interaction data. In conclusion, the above strategies offer helpful analytical solutions for restructuring data collected with different instruments across multiple studies to the same format. Further, the DREAM BIG model of harmonization and replication permits the following questions about the impact of parenting on development across the lifespan to be addressed: (i) What aspects of parental care are most important?; (ii) During what phase of development?; (iii) Which children are at highest risk?; and (iv) For what kind of outcomes?

## Data Availability

The original contributions presented in the study are included in the article/**Supplementary Material**, further inquiries can be directed to the corresponding author.
