# Supplementary material for: The DREAM BIG project as a model for harmonizing early measures of parental care and parent-child interactions across epidemiological cohorts
Source: Front Child Adolesc Psychiatry. 2023 Oct 20;2:1206922. doi: 10.3389/frcha.2023.1206922 (PMC11732133; doi:10.3389/frcha.2023.1206922)
Supplement: Supplementary file 1 [file Table1.docx]

**The DREAM BIG project as a model for harmonizing early measures of parental care and parent-child interactions across epidemiological cohorts.**

**Supplemental material**

**Supplementary Table S1**. Summary of main findings to date from the DREAM BIG consortium.

|  |  |  |  |  |
| --- | --- | --- | --- | --- |
|  |  |  |  |  |
| **Study** | **Journal** | **Year** | **Combined N** | **Main DREAM BIG findings** |
| Sallis et al. | *JCPP* | 2019 | *N*=19,966 | Cross-cohort replication within DREAM BIG of a latent bifactor structure of child general psychopathology (4-8 years) including a general psychopathology latent factor and specific internalizing and externalizing latent factors. |
|  |  |  |  |  |
| Szekely et al. | *JAACAP* | 2021 | *N*=19,896 | Cross-cohort replication of a latent bifactor structure of prenatal maternal mood and anxiety symptoms, including a general prenatal maternal affective symptoms latent factor and specific factors for general anxiety and depressive symptoms, somatic complaints and pregnancy-specific worries. In meta-analyses of the DREAM BIG cohorts, both the prenatal general affective symptoms factor and pregnancy-specific worries factor were independently associated with the child general psychopathology factor (4-8 years). The prenatal general affective symptoms factor was further associated with the child specific internalizing factor. There were no associations with specific externalizing problems. |
|  |  |  |  |  |
| Neumann et al. | *JCPP* | 2022 | *N*=9,247 | Seven of the 16 tested polygenic risk scores for common psychiatric problems and psychological traits were associated with the child general psychopathology factor (4-8 years) after multiple testing adjustment, two with specific externalizing and five with specific internalizing psychopathology. Most polygenic scores associated with a specific psychopathology factor were also associated with child general psychopathology factor, indicating non-specificity of polygenic risk scores. |
|  |  |  |  |  |
